# In Memoriam

**Published:** 1877-09

**Authors:** 


IN MEMORIAM.—It becomes our painful duty to announce the death of Dr. J. F. Bozeman, one of the most intelligent members of the medical profession in Georgia.

Although not actively engaged in the duties of our calling, for several years past, his connection with the local Society of the city, and his free communication with practitioners here, have been most pleasant and profitable. By the state government, as well as the medical profession, his loss will be sadly felt, and his death seriously deplored.

